# Juçara Fruit (*Euterpe Edulis* Martius) Valorization Combining Emergent Extraction Technologies and Aqueous Solutions of Alkanediols

**DOI:** 10.3390/molecules28041607

**Published:** 2023-02-07

**Authors:** Bruna P. Soares, Ana M. Ferreira, Marina Justi, Luiz Gustavo Gonçalves Rodrigues, J. Vladimir Oliveira, Simão P. Pinho, João A. P. Coutinho

**Affiliations:** 1 CICECO—Aveiro Institute of Materials, Department of Chemistry, University of Aveiro, 3810-193 Aveiro, Portugal; 2 Laboratory of Thermodynamics and Supercritical Technology (LATESC), Department of Chemical and Food Engineering (EQA), Federal University of Santa Catarina (UFSC), Florianópolis 88040-900, Brazil; 3 Centro de Investigação de Montanha (CIMO), Instituto Politécnico de Bragança, Campus de Santa Apolónia, 5300-253 Bragança, Portugal; 4 Laboratório para a Sustentabilidade e Tecnologia em Regiões de Montanha, Instituto Politécnico de Bragança, Campus de Santa Apolónia, 5300-253 Bragança, Portugal

**Keywords:** pressurized liquid extraction, ultrasound-assisted extraction, 1,2-alkanediols, glycerol ethers, anthocyanins, product formulation

## Abstract

Anthocyanins from juçara fruits were extracted by pressurized liquid extraction (PLE) or ultrasound-assisted extraction (UAE), using aqueous solutions of 1,2-alkanediols and glycerol ethers as biobased solvents. The PLE (100 bar, 13 min, 1 mL/min flow rate) in the optimal extraction conditions originated 23.1 mg_anthocyanins_·g_dry biomass_^−1^. On the other hand, the UAE was 10 min long, and the optimal conditions using 1,2-propanediol were 42.6 wt%, 160 W, and pH 7.0, leading to 50 mg_anthocyanins_·g_dry biomass_^−1^. Extractions at the UAE optimized conditions, with aqueous solutions of five different 1,2-alkanediols and three glycerol ethers were performed, and compared to water and ethanolic extracts. The biobased solvent solutions presented anthocyanin yields up to 33% higher than water, and were shown to be as efficient as ethanol/water, but generated extracts with higher antioxidant capacity. The anthocyanin-rich extract of juçara, obtained with 1,2-propanediol, was used in the production of a natural soap and incorporated into a cream, showing that the addition of the juçara extract resulted in an antioxidant capacity in both products.

## 1. Introduction

Juçara (*Euterpe Edulis* Martius) is a tropical palm tree of the Atlantic Forest that produces a spherical purple fruit popularly known as juçara, a by-product of palm-heart extraction from the palm tree [1,2]. The valorization of the juçara fruit contributes to environmental sustainability as well as the recovery and conservation of the Atlantic Forest biodiversity, since it is not necessary to cut the tree to harvest the fruits, which occurs in the extraction of the palm heart, and thus threatens the palm tree with extinction [2,3,4]. The valorization of juçara fruits generates an income source for local communities, employing collectors and providing a work base for handicrafts (leaves, seeds, rachillas, pigments, etc.), food products (juice, frozen pulp), cosmetics, and even medicinal products [1,5].

Juçara fruit is considered as a “superfruit” due to its highly nutritional value and its richness in phenolic compounds, such as anthocyanins and phenolic acids [6]. With a high concentration of the anthocyanins, cyanidin-3-O-rutinoside and cyanidin-3-O-glucoside, these are the main constituents of the phenolic juçara fruits’ profile (around 73% of the total phenolic compounds content) [7]. Anthocyanins are associated with the intense dark purple color of the juçara fruit, and one of their main advantages is their antioxidant properties, making them valuable compounds to the food and pharmaceutical industries [6,7,8]. Regarding the phenolic acid profile, hydroxybenzoic (gallic, protocatechuic), p-hydroxybenzoic (vanillic, syringic), and hydroxycinnamic (chlorogenic, caffeic, p-coumaric, sinapinic, and ferulic) are some of the main phenolic acid constituents [6,8]. However, phenolic compounds, such as anthocyanins, are easily degradable, and bioconverted into phenolic acids by temperature, light, solvents, and pH changes, affecting their bioavailability [9,10,11]. Aiming to overcome these limitations, the use of innovative and environmentally-friendly extraction technologies (reducing time, energy, and solvent consumption), such as pressurized liquid extraction (PLE) and ultrasound-assisted extraction (UAE), have been applied. These technologies have been explored in a wide range of valuable compounds found in low-cost natural products [12,13,14,15].

PLE employs solvents at high pressure and temperature. The potential of PLE is correlated to (i) facilitate the disruption of analyte-matrix by exerting pressure, (ii) increase the solubility, and (iii) improve the mass transfer with the use of liquid solvents at temperatures above their atmospheric boiling point [15,16]. On the other hand, UAE is a low-pressure green extraction technology that involves the use of ultrasounds, which mechanically increase the contact between solvents and natural matrices, allowing for the disruption of the plant cell wall in the case of plant matrices, and thus decreasing the mass transfer resistance [14,17]. For UAE, the cavitation generated by the collapse of a bubble in a liquid is an important driving force in the extraction process, since the cavitation results in extremely high-speed microjets of solvent that reach the vegetal matrix, facilitating the penetration of the solvent for the swelling and solvation process [12,13].

The choice of solvent and temperature is crucial for both extraction methods. Differences in temperature and solvent composition change the solvents’ dielectric constant (ε_0_), and thus their polarity [18]. High temperatures destroy the hydrogen-bond structures of water, reducing its polarity and allowing for a better dissolution of polar and non-polar substances [16,18]. Organic solvents, such as ethanol and methanol, are commonly used extraction solvents, and present lower dielectric constants than water [19]. In this perspective, it is possible to combine the polarity of organic solvents and water to generate environmentally aqueous solutions with the desired polarity by changing the concentration of the solvent and the applied temperature. Amphiphilic compounds, such as alkanediols and glycerol ethers, can offer this desired tunable polarity and can be a worthwhile opportunity to enhance the performance and extend the applicability of these emerging extraction methods [20,21,22].

Various compounds have been proposed as solvent additives for subcritical water extraction [23,24], including aqueous solutions of DES for the extraction of anthocyanins from jaboticaba peels using pressurized liquid extraction [25]. Some works propose the use of alkanediols as DES precursors for the extraction and recovery of bioactive compounds from natural sources [26,27,28,29]. Recently, the use of biobased solvents in PLE applications has been expanded. There are reports of studies on the use of limonene [30], glycerol [31], and other renewable biosolvents [32,33]. However, the applications of biobased solvents in the extraction of bioactive compounds combined with alternative extractions have been rarely addressed.

Biobased solvents are produced from renewable resources [34,35]. One of the most studied biobased solvents is the 1,2-propanediol, also known as propyleneglycol, which can be produced from de-oxy sugars using microorganisms for its fermentation and through hydrogenolysis of glycerol, a by-product of biodiesel production [34,35,36]. The 1,2-propanediol is considered as a GRAS solvent (Generally Recognized as Safe) for food, cosmetics, and drug applications [37,38]. Its use includes the production of unsaturated polyester resins, as an additive in nutrition products, colorings, and flavoring agents, as well as a component of lubricants and anti-freezing agents in cosmetics [35]. Although alkanediols, such as 1,2-propanediol, are industrial biosolvents and have been adopted for many applications, they have been poorly explored as extractive solvents to date. Recently, Vieira et al. [39] studied glycerol and a series of alkanediols (1,2-ethanediol, 1,2-propanediol, 1,3-propanediol, 1,3-butanediol, 1,2-pentanediol, 1,5-pentanediol, and 1,2-hexanediol) for the extraction of bioactive compounds from *Juglans regia* L. leaves by the heat-assisted method. In this work, the 1,2-alkanediols, 1,3-propanediol, and 1,3-butanediol showed the best results (high phenolic concentration and cytotoxicity potential against cervical carcinoma cells). To the best of our knowledge, the extraction techniques reported in the literature using alkenodiols and glycerol were all carried out at low-pressure and mainly applying high concentrations of the alkanediols from 25 to 100% (*v*/*v*). There are very few works in which alkanediols are applied in UAE [40,41].

This work aims to extract anthocyanins from juçara fruit using aqueous solutions of the 1,2-alkanediols and to apply alternative extraction technologies, such as PLE and UAE. First, a kinetic study using 1,2-propanediol aqueous solutions was carried for PLE and UAE. Then, experimental conditions for each extraction technology, namely, temperature/amplitude, solvent concentration, and pH, were optimized by response surface methodology (RSM). Since the preliminary results showed that the UAE technique provides the highest extraction yields of anthocyanins (in a shorter time and with a lower solid-to-liquid ratio compared to PLE), the screening of several aqueous solutions of 1,2-alkanediols (1,2-ethanediol, 1,2-propanediol, 1,2-butanediol, 1,2-pentanediol, and 1,2-hexanediol) and glycerol ethers (1.0.1), (2.0.2), and (2.0.0), at the optimized conditions by UAE, was conducted concerning the extraction yield of anthocyanins and extracts of antioxidant activity. Finally, to demonstrate the potential of the anthocyanin-rich extract in cosmetic products, the extract obtained by UAE using 1,2-propanediol under the optimized conditions was directly used as an additive to produce natural soap and incorporated in a cream formulation.

## 2. Results and Discussion

### 2.1. Optimization of PLE Extraction Conditions

The extraction kinetics were first established at 10 MPa, 80 °C, 1 mL/min, 30 wt% of 1,2-propanediol concentration, and pH 4.5 to enable the definition of the extraction time, and then, this time was applied for all assays and solvents studied. The overall extraction curve (OEC) representing the change in the yield of extraction (mg_anthocyanins_·mL^−1^) with the extraction time is presented in Figure S1 (Supplementary Materials).

The analysis of the kinetics behavior for PLE from juçara extracts showed the following mass-transfer mechanisms: constant extraction rate period (CER), falling extraction rate period (FER), and diffusion-controlled period (DC). In addition, the extraction time was fixed at 13 min for all assays, as this represents the beginning of the DC period where most of the soluble anthocyanins were already extracted (after 13 min, the yield increment was less than 3%).

All PLE assays were performed according to the CCRD plan, and considering the fixed conditions of pressure at 10 MPa, flow rate of 1 mL/min, and time of 13 min for the recovery of extract samples rich in anthocyanins. The extracts were analyzed according to the yield of extraction (mg_anthocyanins_·g_dry biomass_^−1^), and the results are presented in Table 1.

The model was tested at 10% significance level. From the Pareto chart, obtained before the successive adjustments (Figure S2 in the Supplementary Materials) the three variables were found to be significant for the extraction efficiency (*Y*) of anthocyanins, namely, *x*_1_ (temperature), *x*_2_ (C_alkanediol_), and *x*_3_ (pH); the quadratic effect of both *x*_2_ (C_alkanediol_) and pH and the interaction between temperature and pH. After excluding the parameters that were not statistically significant from the model, the linear correlation of *x*_2_ was also not included and the final model obtained is described in Equation (1):

(1) $$Y=1.24*10^{1}+5.88*10^{-2}x_{1}+3.20*10^{-3}x_{2}^{2}-3.14x_{3}+5.99*10^{-1}x_{3}^{2}-2.98*10^{-2}x_{2}x_{3}$$

**Figure 1:**
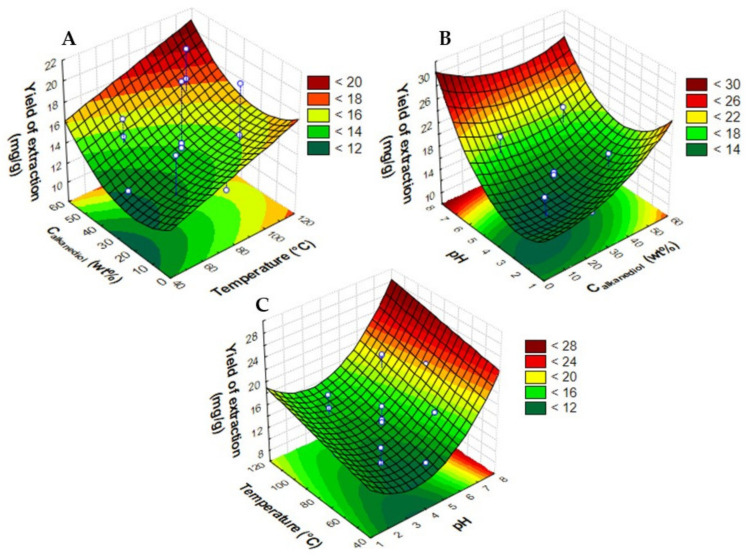
Response surface plots obtained for the CCRD (2^3^) applying PLE regarding the alkanediol concentration (Calkanediol in wt%), temperature (°C), and pH in terms of yield of extraction of anthocyanins (mg_anthocyanins_·g_dry biomass_^−1^). (**A**) C_alkanediol_ and Temperature, (**B**) pH and C_alkanediol_, and (**C**) Temperature and pH.

The regression coefficients and statistical parameters for the model can be seen in Table S1 in the Supplementary Materials. This model presents a R^2^ = 0.81, and the F test confirms the global significance of the model (*p*-value, 1.89 × 10^−6^). According to the proposed model and three-dimensional (3D) response (see Figure 1), the temperature (*x*_1_) has a positive effect on the anthocyanins extraction, although pH (*x*_3_) is the most significant variable. From Table 1, it is also evident that by maintaining the same temperature and concentration conditions, higher increases in extraction yield were found for pH 6.0 compared to pH 3.0. Although in the literature it is accepted that lower pH favors the stability of anthocyanins, generally under acidic conditions, anthocyanins exist in the form of flavylium cation, which shows lower degradation [42]. In this work, pH around the neutrality favors the extraction of anthocyanins by alkanediols.

The results depicted in Figure 1 show that for PLE, the extreme upper values in alkanediol concentration, temperature, and pH present the highest yield of anthocyanins. In fact, the predicted values from the model show that aqueous solutions of 1,2-propanediol at 55.2 wt%, 114 °C, and pH around 7.0, provide the highest yield of extraction of anthocyanins. The accuracy of the model was checked by an experimental validation test at the optimum operational conditions with a low deviation (6.2%) compared to the predicted value (experimental: 23.11 ± 0.06 mg_anthocyanins_·g_dry biomass_^−1^; predicted: 24.63 mg_anthocyanins_·g_dry biomass_^−1^). The optimal conditions coincide with the most extreme conditions used experimentally, which were chosen considering restrictions that must be taken into account: (i) higher concentrations of 1,2-propanediol increase the solvent viscosity to a level not supported by the PLE pump, and (ii) high temperatures accelerate the thermal degradation of anthocyanins by oxidation, hydrolysis, and polymerization reactions [43].

In this work, the highest temperature tested (114 °C) was the optimal condition for extraction of anthocyanins from juçara pulps. Different results were obtained by Garcia-Mendoza et al. [44], i.e., the highest anthocyanins yield (1.76 ± 0.09 mg·g dr ^−1^) in PLE was obtained from juçara residues at 40 °C using acidified water, since at higher temperatures (60 and 80 °C) the authors report a reduction in the anthocyanins content. On the contrary, in the work of Benvenutti [25], the highest content of anthocyanins from jaboticaba peels by PLE, and using DES aqueous solutions, was found at temperatures around 90 °C.

The differences observed in these works may be related to the solution chemistry and kinetics of dissolution. For instance, in the case of the predominant flavylium cation, cyanidin-3-O-glucoside, a highly water-soluble form (due to the structure moieties of sugars) is found until pH values around 4.0 [45], but as the pH rises, the flavylium cation gives rise to the prevalent quinoidal form. Torskangerpoll and Andersen [46] studied the changes in color, solubility, and stability regarding the pH range for three anthocyanins including cyanidin-3-O-glucoside, and they showed different times of dissolution for this specific anthocyanin at different pH buffer solutions: At pH around 5.0, 2 days were necessary for complete dissolution; at pH from 6.0 to 6.9, 1 day was required; and at pH 7.2, it took only 1 h.

In this work, for PLE extraction, the neutral pH was the most appropriate for anthocyanins solubilization and extraction, considering the short extraction time of 13 min. At the end of the extraction, the following optimum conditions were applied (114 °C, pH 7.0, and 55.2 wt% of alkanediol). Moreover, the pH of the extracts was measured and hovered to 4.5, which shows that the biomass, containing a high concentration of anthocyanins, acidified the extract. In fact, the pH in juçara fruit and pulp is around 4.5 to 5.6 [2], acidifying the final extract. For comparison, three replicas of the optimal conditions, but changing the pH from 7.0 to 2.0, were performed, and the extraction response was lower (15 ± 2 mg_anthocyanins_·g_dry biomass_^−1^), as expected. The pH of the final extracts for these last assays was 4.0, which indicates that the biomass increased the pH of the alkanediol solution during the extraction. It is important to note that in the work of Torskangerpoll and Andersen [46], an increase in the pH of only 0.3 was sufficient to decrease the time of dissolution of cyanidin-3-O-glucoside from 1 day to 1 h, which is probably a result of different forms of anthocyanins in equilibrium.

Since the concentration of alkanediol was not one of the most significant variables for the anthocyanin extraction by PLE, contrary to the pH, which was the most important factor, the range of pH evaluated for UAE was extended (from 1.9 to 12.0), maintaining the same alkanediol concentrations studied for the PLE and evaluating the effect of temperature through changes in the percentage of the system power (amplitude).

### 2.2. Optimization of UAE Extraction Conditions

For UAE, the optimization of the extraction conditions was performed considering amplitude (x_1_), corresponding to a percentage of the power of the ultrasonic probe (400 W), evaluated from 10–50%, the concentration of the 1,2-propanediol (x_2_, C_alkanediol_) in the same range used with PLE (from 4.8 to 55.2 %wt) and pH (x_3_), evaluated from 1.9 to 12.0 based on a CCDR (2^3^). The time was set to 10 min according to preliminary kinetic tests (see Figure S3 of Supplementary Material). For method comparison, the response used in the predictive model was the same as PLE optimization, the yield of extraction of anthocyanins (expressed in mg_anthocyanins_·g_dry biomass_^−1^). The yields of extraction of both anthocyanins are shown in Table 2, along with the corresponding extracting conditions.

As obtained from the Pareto chart (Figure S4, Supplementary Materials), linear and quadratic forms of *x*_1_ (amplitude), *x*_2_ (C_alkanediol_), and *x*_3_ (pH) are significant to represent the extraction yield (*Y*). Following the usual procedure of successive fittings to ignore the non-significant statistical variables, the model is described by Equation (2):

(2) $$Y=2.73*10^{1}+8.27*10^{-1}x_{1}-1.05*10^{-2}x_{1}^{2}+1.59*10^{-1}x_{2}-1.94*10^{-3}x_{2}^{2}+1.60x_{3}-1.39*10^{-1}x_{3}^{2}$$

**Figure 2:**
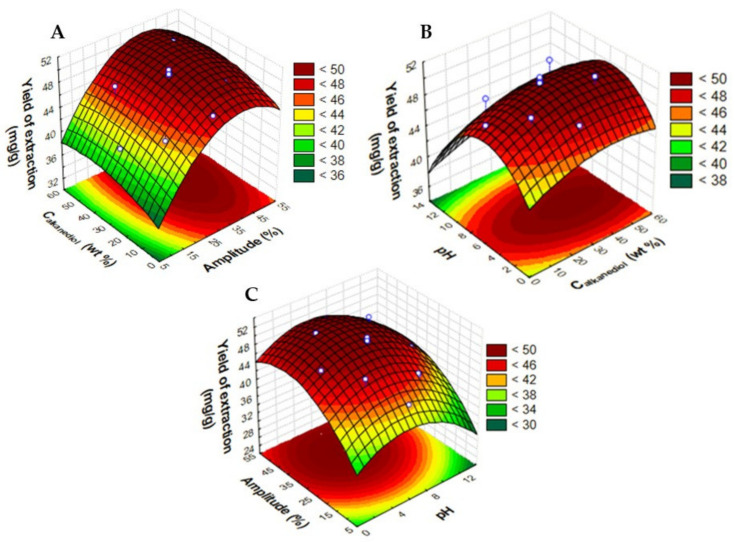
Response surface plots obtained for the CCRD (2^3^) regarding the application of UAE, considering the alkanediol concentration (C_alkanediol_ in wt%), amplitude (%), and pH in terms of yield of extraction of anthocyanins (mg_anthocyanins_·g_dry biomass_^−1^). (**A**) C_alkanediol_ and Amplitude, (**B**) pH and C_alkanediol_, and (**C**) Amplitude and pH.

Table S2 (Supplementary Materials) compiles statistical information on the regressed model. This model presents R^2^ = 0.95, and the F test confirms the global significance of the model (*p*-value, 9.36 × 10^−7^). According to Equation (2) and the three-dimensional (3D) response surfaces depicted in Figure 2, the amplitude (*x*_1_) increases the extraction yield up to 40% of x_1_, the alkanediol concentration (*x*_2_) to around 40 wt%, and the pH (*x*_3_) until 6.0. This trend of the model makes intermediate values of pH, concentration, and amplitude to be considered as the optimal extraction values, as depicted by the curvature profile of the response surfaces in Figure 2. In the case of UAE, aqueous solutions of 1,2-propanediol at 42.6 wt%, 40% of amplitude, and pH around 7.0 provide the highest yield of extraction of anthocyanins (the experimental value is 50 ± 1 mg_anthocyanins_·g_dry biomass_^−1^). The predicted value is (51.18 mg_anthocyanins_·g_dry biomass_^−1^) showing the very good accuracy of the model with a deviation to the experimental value close to 1.4%. Although there are differences between the variables using the two extraction methodologies (UAE or PLE), it is notorious that the yield of extraction obtained for the UAE is twice superior to the yields obtained by PLE, although the solid-liquid ratio used for UAE was lower (1:50 g/mL) than for PLE (1:13 g/mL)

As previously mentioned, the pH of the solvent is an important factor for anthocyanins stability and solubility, and consequently for its extraction from juçara. For UAE, the solvent pH around 7.0 is the optimal condition to extract anthocyanins from juçara fruit, and the final pH value of these extracts was around 4.5, in line with what was observed for PLE. Surprisingly, when the optimal point was repeated, but the pH changed from 7.0 to 2.0, the final pH value of the extracts was around 2.5, which was more acidic than PLE (final pH = 4.0). Moreover, the yield of extraction for this last assay at pH 2.0 is (48 ± 1 mg_anthocyanins_·g_dry biomass_^−1^), which is considerably closer to the optimum UAE (pH 7.0 optimized value 50 ±1 mg_anthocyanins_·g_dry biomass_^−1^) than PLE (pH 7.0 optimized value 23.11 ± 0.06 mg_anthocyanins_·g_dry biomass_^−1^; pH 2.0 optimized value 15 ± 2 mg_anthocyanins_·g_dry biomass_^−1^) which reinforces that for UAE, the variable with the most significant impact is amplitude and not pH.

For UAE, the amplitude is correlated with two main factors: Cavitation and temperature. Ultrasounds incite the formation of small bubbles in a cavitation phenomenon, which leads to the breaking of the juçara cell wall, rapidly increasing the local temperature in the system. Therefore, higher ultrasonic amplitude generates a higher number of cavities, increasing the mass transfer and allowing for better penetration of the medium, enhancing the extraction yield of anthocyanins [13,17]. For each assay, the initial and final temperatures were measured (Table S3 in the Supplementary Materials). While the initial temperature was always around room temperature, the final temperature (after 10 min) for almost all the amplitudes was between 92.2 and 99.1 °C, except for 10% of the amplitude. From these values, it is possible to notice that temperature and amplitude do not have a linear correlation despite being closely related. The temperature rise gradient for larger amplitudes is higher, although the final temperatures are similar, increasing the extraction yield. As it is possible to note in Table 2, for the same pH and alkanediol concentration, higher yields of extraction were found for the amplitude of 42% compared to 18%, despite the fact that the final temperature difference was only about 5 °C (Table S3 in the Supplementary Materials). High-rise gradients of temperature decrease the solvents’ viscosity and surface tension, facilitating their penetration into the biomass matrix.

Regarding the solvent concentration until 42.6 wt%, the increased alkanediol proportion reduces the solution’s polarity, favoring the interaction energy between the hydrophobic and hydrophilic target compounds and the solvent molecules [16,18]. In other works, concerning the extraction of anthocyanins from juçara pulps/residuals, concentrations around 50–70% (*v*/*v*) of ethanol exhibit higher anthocyanin yields, suggesting that the intermediate concentration of organic solvents is the best condition for the recovery of anthocyanins [44,47,48].

In summary, in addition to the effects of pH (on anthocyanin stability) and solvent type and concentration (on extraction yield), the specific conditions of each extraction methodology should also be considered when extracting anthocyanins. While temperature and pressure (up to 10 MPa [16]) are the most important parameters for PLE, power, intensity, and frequency are the most important parameters for UAE, which are all related to amplitude. In addition, temperature has a strong influence on UAE extraction, as it can lead to altered solvent properties and has sonochemical effects related to cavity bubble collapse [49]. Note that in the case of UAE, temperature is also an intrinsic parameter related to amplitude. The results obtained in this work support all these facts, since temperature and amplitude are significant variables for PLE and UAE, respectively.

### 2.3. Solvent Effect on UAE Recovery of Anthocyanin-Rich Extract

**Figure 3:**
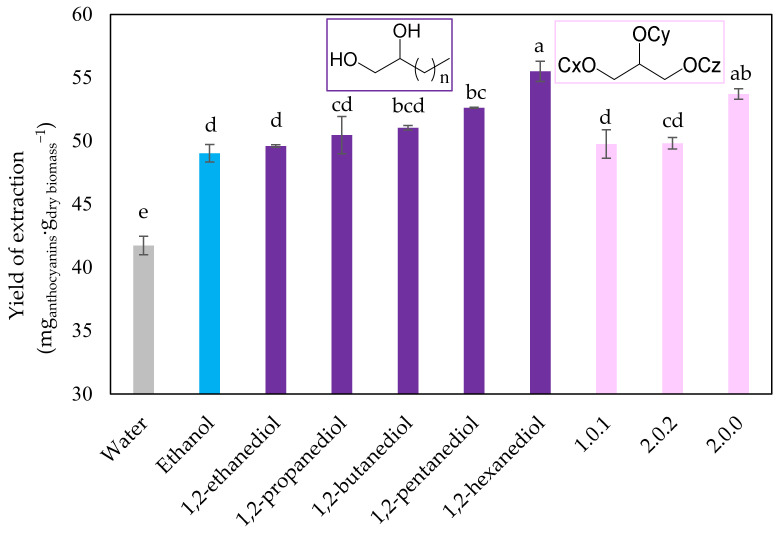
Comparison of different solvents in the extraction of anthocyanins (in terms of yield of extraction in mg_anthocyanins_·g_dry biomass_^−1^) and their corresponding standard deviations. Purple bars represent the 1,2-alkanediols, and light pink bars represent the glycerol ethers studied. Different letters represent statistically different values (*p* ≤ 0.10), while the same letters represent statistically equal values (*p* > 0.10) by the Tukey test from ANOVA.

Subsequently, anthocyanin-rich extracts were obtained by UAE at the optimum extraction conditions established in the previous section using different solvents, namely, the 1,2-alkanediol series (1,2-ethanediol, 1,2-propanediol, 1,2-butanediol, 1,2-pentanediol, and 1,2-hexanediol), the glycerol ethers (1.0.1), (2.0.2), and (2.0.0), as well as water and ethanol, as control. The judicious choice of the alkanediols in question was based on three particularities of these solvents: (i) Their cost, (ii) applicability, and (iii) previous experience [50,51]. The 1,2-alkanediols and glycerol ethers were shown to be good solvents for the solubility enhancement of phenolic compounds. The results regarding the yield of extraction, the total phenolic content (TPC) and antioxidant activity by ABTS are summarized in Figure 3 and Table 3, respectively.

The yield of extraction ranged from 41.8 to 55.5 mg_anthocyanins_·g_dry biomass_^−1^ with the highest value obtained for 1,2-hexanediol (33% higher than water), followed by the glycerol ether (2.0.0) (28.7% higher than water). The results reported in Table 3 and Figure 3 suggest that increasing the alkyl chain size of alkanediols increases the yield of extraction of anthocyanins, due to the change in the polarity of the solvent system that favors the solubilization. Although anthocyanin solubility studies are scarce, mainly due to their high commercial value in high purities, the study of other model molecules (also presented in juçara fruit pulps), such as phenolic acids, indicates that increasing the alkyl chain size of alkanediols favors the solubilization of these compounds [50]. As previously mentioned, anthocyanins are generally soluble in water. However, the polyphenolic structure adds a measure of hydrophobic character, which gives relevance to organic solvents. The combination of the nature of these polar and non-polar anthocyanins makes the alkanediol/water mixture a good solvent for their solubilization, since alkanediols also exhibit an amphiphilic structure.

Regarding TPC, the highest values were found for 1,2-butanediol, 1,2-pentanediol, and 1,2-hexanediol alkanediols and the symmetric glycerol ethers (1.0.1) and (2.0.2) (being 20–25% higher than in water). This is probably a consequence of the extraction of more apolar phenolic compounds, such as phenolic acids and flavonoids. To evaluate the functional properties of the juçara pulp extracts, the antioxidant activity was assessed by ABTS, and the results showed that the extracts which exhibit the best antioxidant capacity were those from 1,2-propanediol and 1,2-hexanediol. In this case, the glycerol ether extracts presented similar ABTS values as the ethanolic ones.

These results suggest that both families of solvents tested, alkanediols and glycerol ethers, have a higher ability to extract anthocyanins than water and ethanol, solvents commonly used to extract these target compounds. The 1,2-hexanediol presented a higher yield of extraction of anthocyanins and selectivity, since even other alkanediols can extract with a similar yield of phenolic compounds (regarding TPC values). Moreover, 1,2-hexanediol generates extracts with higher concentration of anthocyanins and good antioxidant capacity.

Although it is well established in the literature that additional acidic pH is more suitable for anthocyanin extractions, the results presented in this work suggest that when using alkanediols, a pH close to 7.0 can generate extracts with higher anthocyanin extraction yields than those with a more acidic pH. In terms of the process, this is an advantage of the alkanediols, since the aqueous solutions of these compounds in the optimized concentration (46.2 wt%) present a pH close to neutrality, which eliminates the pH adjustment step (see Table S4 in the Supplementary Materials).

In addition, depending on the application, alkanediols are ingredients that offer advantages to the final product (at a regulated concentration by safety assessments), as in the case of cosmetics, in which short-chain alkanediols, such as those studied in this work, confer properties, such as viscosity reducers, skin conditioning, and humectants, and are part of the chemical solvent constitution of cosmetics. This is an advantage over volatile organic compounds (VOCs), such as ethanol, methanol, or acetone, as these alkanediols may be retained in the extract (at specific concentrations) depending on the final application.

At this point, the extracts obtained using 1,2-propanediol (propylene glycol) reached the highest antioxidant activity by ABTS, which is a great advantage of this compound over the other alkanediols studied, since statistically, the values obtained for the extraction yield were similar between 1,2-propanediol, 1,2-butanediol, and 1,2-pentanediol (see Figure 3). The biggest advantage of 1,2-propanediol compared to others is that this solvent is considered GRAS. In addition, it is widely used in cosmetic formulations, which makes the obtained anthocyanin-rich extracts potential ingredients for cosmetic application with a high content of antioxidant compounds.

### 2.4. Characterization of Formulations Containing the Juçara Extract

To demonstrate the potential of using the anthocyanin-rich extract from juçara in cosmetic product formulations, this extract was used as an additive in the manufacture of natural soap and natural cream. The extract used in these products was the final juçara extract obtained by UAE under optimized conditions (42.6% 1,2-propanediol, pH 7.0, at 160 W for 10 min). Juçara soap (JB) was prepared using the final juçara extract as an additive, without additional polishing or purification steps. On the other hand, juçara cream was formulated with the final juçara extract (JC), but also with the final concentrated juçara extract (CJC).

**Figure 4:**
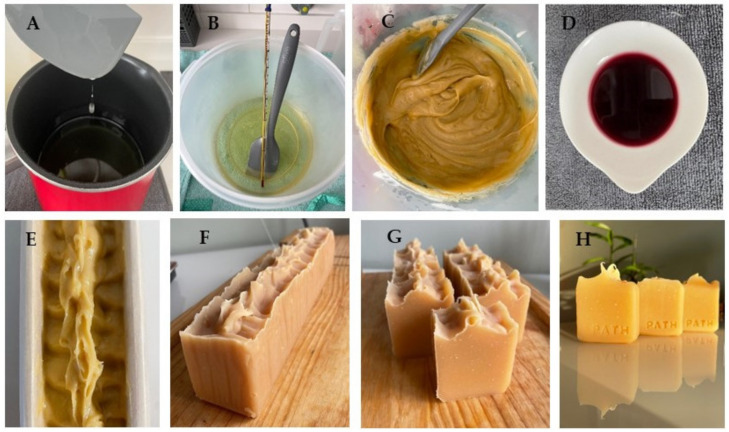
Juçara natural soap production. (**A**) Oil and butter heating; (**B**) melted butter and oil mixture; (**C**) soap mass before additives; (**D**) juçara extract (42.6% of 1,2-propanediol in 1:50 g/mL of juçara dried pulp); (**E**) parchment paper-lined molds filled with soap mass; (**F**) loaf of soap after 24 h; (**G**) soap bars; and (**H**) soap bars after cure.

The production scheme of juçara natural soap is shown in Figure 4. After 1 week of curing, the pH of the soaps (JB and BB) was measured and the final pH for both samples was about 9.5. The acceptable pH range for natural soaps is between 8.0 and 10, since a pH above 10 expresses a high percentage of unknown and unsaponifiable substances due to incomplete alkaline hydrolysis. In addition, the high alkalinity of soaps destroys the protective acid mantle of human skin, which acts as a protective barrier against the penetration of microbes. On the other hand, the use of a weakly-alkaline soap is less harmful to the skin and favors detergent properties [52].

Due to the antimicrobial and antifungal effects of essential oils, cosmetic preparations, such as natural herbal soaps, do not necessarily require an additional chemical preservative if they contain an essential oil or a single compound as an active agent [53], such as rosemary oil, or, in the case of this work, juçara extract. The antioxidants present in essential oils and juçara fruits are free radical scavengers that protect soaps and human skin from oxidative stress, and assist in preventing the decomposition of fats or oils by oxidation [54].

**Figure 5:**
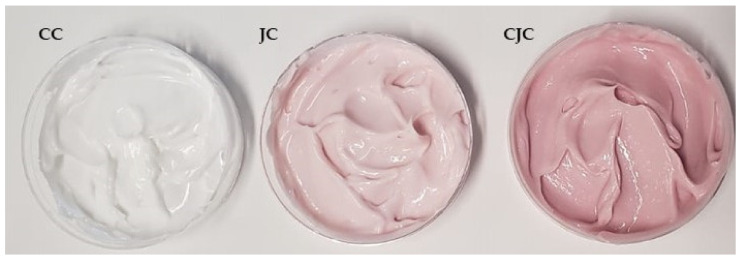
Formulation creams evaluated in this work. (CC) Control cream; (JC) juçara cream; and (CJC) concentrated juçara cream.

Regarding the three creams studied (Figure 5), the pH of each cream was about 4.0, which corresponds to an acceptable pH for topical application (between 4.0 and 6.0) [55]. In addition, the pH values obtained show that the juçara extracts do not alter the final pH of the creams.

Table 4 shows the antioxidant activity by ABTS for the soaps and creams produced. Comparing the two soaps, the antioxidant activity by ABTS method is 2.4 times higher for JB than BB. It is important to mention that in the formulation of both soaps (BB and JB), essential oil additives with antioxidant activity were incorporated, as confirmed by the ABTS value found for BB = 6.6 μmol_TE_**·**g_formulation_^−1^. For the creams, the antioxidant activity for the control was around zero, since the cream base formulation did not contain antioxidants as additives. On the other hand, the addition of juçara extracts in the cream formulation resulted in an increase in antioxidant activity, with the concentrated juçara cream (CJC) showing 2.2-fold antioxidant activity compared to juçara cream (JC), which indicates that for creams, concentrating the juçara extract by 50% also increases the antioxidant activity of the final creams by around 50%. In conclusion, the use of juçara extracts as an additive in cosmetic products (soap and cream) leads to an increase in antioxidant activity.

Cream formulations using bio-propylene glycol rather than commercial propylene glycol in UAE extractions were also performed, in order to evaluate the direct applicability of a solvent from renewable sources. Without surprise, similar levels of antioxidant activity were found by ABTS, suggesting that bio-propylene glycol-based extracts can also be used as a formulation ingredient for the production of 100% natural cosmetic products. It is important to note that propylene glycol is widely used as an ingredient for cosmetic formulations, including the production of soaps and creams, acting as a humectant, skin-conditioning agent, viscosity reducer, and as a fragrance ingredient [35,38]. In this sense, the use of juçara anthocyanin-rich extracts promotes both antioxidant activity and improvement of cosmetic formulations through the inherent physical-chemical properties of the solvent.

## 3. Material and Methods

### 3.1. Raw Material

The juçara fruit (*Euterpe Edulis* Martius) pulps were produced and kindly supplied by Duas Rodas company (Jaraguá do Sul (SC), Brazil), harvested in June 2020, in Garuva, Jaraguá do Sul and Joinville (SC), Brazil. The pulps were pasteurized, providing a shelf life of 36 months under refrigerated conditions (−18 °C). The juçara pulp samples were frozen in 5.0 kg packages at the Laboratory of Thermodynamics and Supercritical Technology (LATESC, UFSC, Brazil). Frozen portions of the sample were freeze-dried (Liotop, model LD101, São Paulo, Brazil) for 48 h in order to reduce the moisture content from approximately 96% to 4–6%, as determined by the 012 IV method [56]. Dried samples were ground with a knife mill (average particle diameter = 0.345 mm) and stored in polyethylene bags at −18 °C until further use.

### 3.2. Chemicals

The chemicals used in this work are detailed in Table 5, along with their CAS, sources, and mass purities. Water was double distilled, passed across a reverse osmosis system, and further treated with a Milli-Q plus 185 water purification apparatus. The glycerol ethers used were synthesized as described in detail in a previous work [51]. All oils, butter, essential oils, and sodium lactate for soap production were purchased from Jabonarium. The Versatile™ base cream was purchased from Fagron Iberica S.A.U. (Barcelona, Spain). Bio-propylene glycol from Orlen was kindly supplied by Brenntag Portugal.

### 3.3. Extraction Methodologies

The attainment of the anthocyanin-rich extracts from juçara dried pulps was performed by the combined use of aqueous solutions of alkanediols with emergent extraction techniques, namely, pressurized liquid extraction (PLE) and ultrasound-assisted extraction (UAE).

#### 3.3.1. Pressurized Liquid Extraction

A self-assembled PLE unit was used to recover anthocyanins from juçara dried pulps. The customized apparatus was built to perform subcritical water extractions [57]. The apparatus contains an extraction vessel of 17.3 mL (internal diameter of 9.40 mm and a height of 250 mm) made of 316/316 L stainless steel. The vessel was heated using an electrical heating jacket system, using 250 W resistances (designed to withstand up to 300 °C). A pre-heater system (with two resistances of 250 W with steel coil), brings the solvent up to the operating temperature before entering the extraction vessel.

The temperature of the extraction vessel and the pre-heater was controlled by a digital temperature controller (PXF4 Fuiji electric, Tokyo, Japan). The thermocouple (model ADAM 4019+-AE, Advantech, Irvine, CA, USA) allowed for the acquisition of temperature data using a type-k temperature sensor (model BT.MiK1.2.1, 5/3.15/35/50, Bresimar Automação, Aveiro, Portugal). The extraction pressure was monitored by a digital manometer (model PTI-S-AG250-15AV, Swagelok, London, UK). The solvent was pumped by a compact HPLC pump (PU-4580 Jasco, Easton, MD, USA) into the extraction vessel containing the biomass, where the pressure was controlled by a needle valve (model SS-41GS2, Swagelok, Sauron, OH, USA).

**Figure 6:**
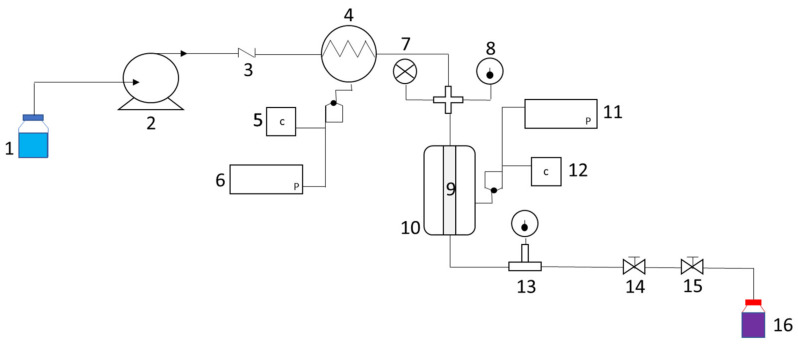
Schematic design of the customized PLE unit: (1) Solvent reservoir; (2) solvent pump; (3) check valve; (4) pre-heater exchanger; (5 and 12) automatic temperature controller; (6 and 11) data acquisition by computer; (7) manometer; (8) thermocouple; (9 and 10) extraction vessel with electrical heating jacket; (13) regulator needle valve; (14) regulator valve; (15) block valve; and (16) amber glass flask.

A schematic diagram of the PLE customized apparatus is illustrated in Figure 6, containing the main constituents of a supplying pump, pre-heating and temperature-controlled system, extraction vessel, and pressure-controlled valve.

The experiments were performed at continuous mode, with pressure fixed at 10 MPa, a flow rate of 1 mL/min, and time of 13 min, which was defined through an extraction kinetic (performed with 1 g of sample, at 80 °C, 30 wt% of 1,2-propanediol solution as the solvent, and pH of 4.5) based on the yield of extraction of anthocyanins (mg_anthocyanins_·mL^−1^). A pressure of 10 MPa was chosen to perform the high-pressure extractions since it has been reported in the literature that a pressure higher than 10 MPa has a negligible effect on the solubilization and recovery of the target compounds [16,57]. One of the explanations for this is attributed to the change in density with pressure, which is generally insignificant due to the non-compressibility of the liquid [58]. In addition, the use of higher pressure conditions is directly associated with higher energy requirements.

After the extractions, the samples were immediately collected in amber flasks, diluted, and quantified by HPLC-DAD on the same day.

#### 3.3.2. Ultrasound-Assisted Extraction (UAE)

UAE was carried out according to the method described by Cheok et al. [59]. The assays were performed using the UAE equipment (Branson 450 Digital Sonifier), where a titanium probe was immersed right below the sample, containing 0.2 g of juçara dried pulps, added to 10 mL of solvent (1,2-propanediol/water mixture) in a sonication time of 10 min, defined from preliminary tests (performed for 30 wt% of 1,2-propanediol at pH 7.0 and 30% of amplitude). After the extractions, the samples were centrifuged for 10 min at room temperature at 10,000 rpm in an Eppendorf centrifuge 5425, collected in amber flasks, diluted, and quantified by HPLC-DAD on the same day. It is important to mention that amplitude affects the amount of power applied during the experiment, where 100% of amplitude in the equipment corresponds to 400 W.

#### 3.3.3. Response Surface Methodology

The central composite rotatable design (CCRD) is a response surface methodology, in which a multivariate statistical tool is applied herein to optimize the extraction conditions of anthocyanins from juçara pulps. The relationship between the response and the independent variables was described according to the following polynomial equation:

(3) $$y=\beta_{0}+\sum_{i=1}^{k}\beta_{i}X_{i}+\sum_{i=1}^{k}\beta_{ii}X_{i}{}^{2}+\sum_{i<j}^{k}\beta_{ij}X_{i}X_{j}$$

where *β*_0_ represents the intercept or regression coefficient, *β_i_*, *β_ii_*, and *β_ij_* are the linear, quadratic, and interaction coefficients, respectively, *X_i_* and *X_j_* are the independent variables, and *k* is the number of variables studied that can influence the response *y*. In this work, the independent variables were subjected to factorial planning of 2^3^ (3 variables, and 2 levels) to optimize the yield of extraction of anthocyanins (mg_anthocyanins_·mL^−1^) as the measured response function. Nineteen experiments were performed, including five replicates at the central point. A 90% confidence level was chosen to analyze the results. To verify the significance of the model parameters, the analysis of variance (ANOVA) was implemented. The coefficient of determination (R^2^) and adequate precision (F calculated value and *p*-value) were used to evaluate the adequacy of the polynomial equation for the response and optimum values. The experimental design, statistical analysis, and regression model were accomplished using Statistica version 13.5.0.17.

In this work, two factorial plannings were executed for both techniques of extraction, PLE and UAE. The first factorial planning was applied for PLE, in which the chosen levels of the independent variables were temperature (60, 80, and 100 °C), 1,2-propanediol concentration in water (15, 30, and 45 wt%), and pH (3.0, 4.5, and 6.0). The second factorial planning was carried out for UAE, in which amplitude (18, 30, and 42%), 1.2-propanediol concentration in water (15, 30, and 45 wt%), and pH (4.0, 7.0, and 10.0) were varied. All coded levels of independent variables used in the factorial planning for the optimization of operating conditions for both PLE and UAE was given in detail in Section 2.1 and Section 2.2, respectively. For both extraction methodologies, the pH of the solutions was adjusted from 0.1 M solutions of HCl or NaOH. 

### 3.4. Anthocyanin-Rich Extracts Characterization

The characterization of both anthocyanins, cyanidin-3-O-glucoside and cyanidin-3-O-rutinoside, was carried out by HPLC-DAD (Shimadzu, model Prominence, Kyoto, Japan). Chromatographic analyses were performed with an analytical C18 reversed-phase column (250 × 4.60 mm), Kinetex 5 μm C18 100 Å, from Phenomenex. The separation was conducted in a gradient system of pure methanol (phase A) and 0.1% of sulfuric-acid-ultra-pure water (phase B). The separation was carried out using the following gradient mode: 0 min 5% of phase A; 1 min 13% of phase A; 2 min 15% of phase A; 3 min 18% of phase A; 3.5 min 20% of phase A; 4 min 25% of phase A; 4.5 min 27% of phase A; 6.5 min 50% of phase A; 8.5 min 5% of phase A; 20 min 5% of phase A. The flow rate was 1 mL/min with a volume injection of 20 µL, and DAD was set at 520 nm. The column oven was operated at a controlled temperature of 55 °C. Each sample was analyzed at least in duplicate. Calibration curves were prepared using the pure anthocyanins with samples dissolved in 0.1% sulfuric-acid-ultra-pure water. Cyanidin-3-O-glucoside and cyanidin-3-O-rutinoside displayed a retention time of 8.0 and 8.7 min, respectively. The results were expressed in mg of anthocyanin per g of dry biomass (mg**·**g_dry biomass_^−1^). Henceforth, the sum of both anthocyanin concentrations expresses the yield of extraction in mg of anthocyanins per g of dry biomass (mg_anthocyanins_**·**g_dry biomass_^−1^).

### 3.5. Comparing Alkanediol Aqueous Solutions with Other Solvents

To compare different solvents, the UAE technique was chosen given (i) the higher extraction yield of anthocyanin achieved in the optimization compared to PLE, (ii) the shortest extraction time (10 min for UAE and 13 min for PLE) and maintenance between two consecutive experiments (around 5 min for UAE and 1 h for PLE), and (iii) the lower solid-liquid ratio required (1:50 g/mL for UAE versus 1:13 g/mL for PLE). Here, the effect of the type of solvent on UAE yield and antioxidant activity was evaluated using the optimized conditions. The 1,2-alkanediol series (1,2-ethanediol, 1,2-propanediol, 1,2-butanediol, 1,2-pentanediol, and 1,2-hexanediol) and the glycerol ethers (1.0.1), (2.0.2), and (2.0.0) were selected in the following previous studies using these compounds [50,51] and compared with the water and ethanol used as control solvents. The chemical structure of the solvents mentioned above is represented in Figure S5 in the Supplementary Materials. In addition to the anthocyanins yield, defined by HPLC-DAD quantification, the following analyses were conducted to evaluate the total phenolic content and antioxidant activity of the extracts recovered using different solvents.

#### 3.5.1. Total Phenolic Content (TPC)

The TPC of juçara pulp extracts obtained for different solvents (1,2-alkanediol series, glycerol ethers, water, and ethanol) was determined by the Folin–Ciocalteu method, according to Koşar et al. [60], with few modifications. The extracts obtained were initially diluted 10 times in the correspondent extraction solvent. Then, 10 μL of the sample solution, 600 μL of distilled water, and 50 μL of Folin–Ciocalteu reagent were mixed. After 1 min, 150 μL of Na_2_CO_3_ 20% (*w*/*v*) were added, and the volume was completed to 1 mL with distilled water. Aliquots of 300 μL of each sample were transferred to wells in a microplate. After 2 h, at 25 °C in the absence of light, the absorbance was measured at 760 nm in a UV-spectroscopy using a BioTeck Synergy 4 HT microplate reader. The analyses were realized in quintuplicate and the TPC was calculated according to a standard curve prepared with gallic acid. The results were expressed in mg of gallic acid equivalent per g of dry biomass (mg_GAE_**·**g_dry biomass_^−1^).

#### 3.5.2. ABTS Radical Scavenging Assay

The mechanism involved in ABTS^+^ radical (2,2′-Azino-bis (3-ethylbenzothiazoline-6-sulfonic acid) diammonium salt) cation quenching follows an electron transfer mechanism. The ABTS assay of the extracts obtained for different solvents (1,2-alkanediol series, glycerol ethers, water, and ethanol) was realized according to Re et al. [61], with some modifications. First, 7 mM ABTS solution and 2.45 mM potassium persulfate solution were allowed to react (1:1) for 16 h at room temperature to generate the radical ABTS^+^. Then, ABTS^+^ solution was diluted with distilled water until an absorbance of 0.700 (±0.05) at 734 nm. The extracts were diluted 10 to 100 times in the corresponding extraction solvent. Thereafter, 20 μL of each sample solution and 280 μL of ABTS^+^ were added to a microplate. Finally, after 30 min of incubation in darkness, the absorbance was read at 734 nm. The analysis was performed in quintuplicate accompanied by a control (20 μL of diluted solvent, 280 μL of ABTS^+^) and the blank for each sample (20 μL of sample, 280 μL of distilled water). Trolox was used as a reference standard, and the concentrations were calculated from a standard curve. The results were expressed in terms of μmol of Trolox equivalent per g of dry biomass (μmol_TE_**·**g_dry biomass_^−1^) for quintuplicate measurements.

#### 3.5.3. Statistical Analysis

The analysis of variance (ANOVA), followed by the Tukey test, were applied using ORIGIN 2018 to compare the significance of the extraction yields, TPC, and antioxidant activity obtained for the two anthocyanins extracted with the different solvents studied (1,2-alkanediol series, glycerol ethers, water, and ethanol) using a significance level of 90% (*p* < 0.1).

### 3.6. Product Formulations Using Juçara Anthocyanin-Rich Extract as Additive

#### 3.6.1. Natural Soap Production by Hot Process Saponification

The natural soaps were manufactured using 5% of juçara extract and 30% of distilled water (% over the total mass of oils). The soaps were prepared by the hot saponification or hot processing method. Sodium hydroxide (NaOH), or lye, was used as the base. A lye calculator was used to determine the rate of saponification for each oil used to formulate the soap, and it indicated that 15% of NaOH (over the total mass of oils) was required. A mixture of oils, including coconut oil (30%), sunflower oil (25%), castor oil (10%), and karite butter (35%) was heated at 100 °C, then, without heating, sodium lactate (2%) was added and mixed. Immediately, the NaOH aqueous solution was prepared and promptly added to the oil mixture and properly blended to mix the ingredients homogeneously. After the texture changed, the additives were incorporated into the soap: A mixture of essential oils (5%) and juçara extract (5%). Parchment paper-lined molds were filled with the soap mixture and saponified for 24 h at room temperature.

After saponification, each soap loaf was removed from the mold, weighed (approximately 0.75 kg), and dimensions were measured (0.045 × 0.05 × 0.225 m). The loaves were cut into nine identical bars using a commercial wire cutter. Each bar had a similar shape and weight, measuring (0.045 × 0.05 × 0.025 m), and weighing approximately 0.08 kg. The soap bars were placed in small wooden containers and stored at room temperature in a dark room with controlled humidity for 1 week. After curing, the soap shavings were wrapped with aluminum foil paper and maintained at −80 °C for further analysis. The abbreviations used to delineate the natural soaps evaluated in this study are: BB = base bar and JB = juçara bar. For the base, the same recipe was used but without the juçara extract, which was replaced by rosemary oil, a conventional antioxidant for natural herbal soaps.

#### 3.6.2. Incorporation of the Extract in a Cosmetic Cream

Juçara extract, rich in anthocyanins, was incorporated into the cosmetic cream Versatile™ enriched oil in water (O/W) emulsion. The cosmetic cream used as a cream base contains natural lipids with moisturizing and protective properties compatible with the incorporation of a wide range of active ingredients. According to the manufacturer, this base cream contains purified water, natural oil, O/W emulsifiers, emollients, lubricants, pro-liposome, vitamin E, chelating agent, silicone, and preservatives, and is free of fragrances, dyes, parabens, mineral oil, SLS, propylene glycol, and 1,4-dioxane. In the preparation of the formulations, 50 mg of the extract was mixed into 1 g of base cream. Three different cream formulations were evaluated: CC = control cream (without the addition of extract); JC = juçara cream (with direct addition of the final UAE extract); and CJC = concentrated juçara cream (juçara extract was concentrated two times by reducing the water content).

#### 3.6.3. Antioxidant Activity Analysis and pH of Product Formulations

To perform the analyses of antioxidant activity of the studied cosmetic products, 100 mg of each soap or 200 mg of each cream was extracted with 1 mL of acetone:ethanol (1:1 *v*/*v*) for 1 h in a Tryster device (Multi Bio RS-24, Biosan) with an orbital rotation of 50 rpm. The sample was centrifuged at 10,000 rpm for 15 min, and the supernatant was carefully decanted without disturbing the pellet. The supernatant was filtered using syringe filters, and the filtrate was used directly to determine the antioxidant activity of the formulations. The antioxidant activity for the formulations was determined by the ABTS method (Section 3.5.2), and the results were expressed in milligrams of Trolox equivalent per gram of formulation (mg_TE_**·**g_formulation_^−1^). In the case of the soap/cream quantifications, the ABTS^+^ solution was diluted with ethanol rather than distilled water, due to the high level of fat ingredients present in these formulations.

To measure the pH, a mass of 1 g of the final formulation (soap or cream) was diluted in 10 mL of distilled water, and the resulting solution was measured using a pH meter calibrated before each measurement (Seven Excellence, Mettler Toledo).

## 4. Conclusions

The development of new approaches to valorize the juçara fruits is essential for maintaining the Atlantic Forest biodiversity and encouraging innovative ideas for its sustainable use. This work proposes the use of juçara fruit pulp as a rich source of bioactive antioxidant compounds. Therefore, this work aims to study the extraction of phenolic compounds and anthocyanins by two emergent methods (PLE and UAE) using aqueous solutions of biobased solvents, namely, alkanediols and glycerol ethers.

For the valorization of juçara fruits, the optimum PLE and UAE conditions using aqueous solutions of 1,2-propanediol as reference solvent were established. For PLE, the best processing conditions were 55.2 (wt%) of 1,2-propanediol, 114 °C, and pH 7.0 (achieving the extraction yield of 23.11 ± 0.06 mg_anthocyanins_**·**g_dry biomass_^−1^). For UAE, the best conditions were 42.6 (wt%) of 1,2-propanediol, 40% amplitude, and pH 7.0 (yield of 50 ± 1 mg_anthocyanins_**·**g_dry biomass_^−1^). The results found for UAE exceeded twice the yield of extraction by PLE, and this method was selected for a solvent screening with alkanediols and glycerol ethers. For the optimized conditions, 1,2-hexanediol was the best solvent for anthocyanins extraction, presenting extracts with high antioxidant capacity and overcoming the extraction yield with conventional solvents, such as water or ethanol.

In the literature, it is well established that acidic pH is more suitable for obtaining anthocyanin-rich extracts. This work showed that using alkanediols, a pH close to 7.0 confers higher extraction yields, which is an advantage of these compounds, since their aqueous solutions have a pH close to neutrality. In some applications, such as in the cosmetics industry, some alkanediols work as formulation ingredients, which allows for the use of juçara extracts without polishing steps, in contrast to what is expected for the case of volatile organic solvents, such as ethanol. Therefore, alkanediols are green alternatives to replace organic solvents and for juçara fruits valorization.

## Figures and Tables

**Table 1:** Central composite rotatable design (CCRD) for the independent variables considered for the PLE extraction with aqueous solutions of 1,2-propanediol from juçara fruit samples at the pressure of 10 MPa and flow rate of 1 mL/min, as well as the results obtained for the extraction assays in terms of yield of extraction of anthocyanins.

Assay	Temperature (°C)	C_alkanediol_ (wt%)	pH	C_cyanidin-3-O-glucoside_ (mg·g_dry biomass_^−1^)	C_cyanidin-3-O-rutinoside_ (mg·g_dry biomass_^−1^)	Yield of Extraction (mg_anthocyanins_·g_dry biomass_^−1^)
1	60 (−1)	15 (−1)	3.0 (−1)	2.73	9.62	12.35
2	100 (+1)	15 (−1)	3.0 (−1)	3.52	12.08	15.60
3	60 (−1)	45 (+1)	3.0 (−1)	3.40	11.51	14.91
4	100 (+1)	45 (+1)	3.0 (−1)	4.06	13.82	17.88
5	60 (−1)	15 (−1)	6.0 (+1)	3.62	12.94	16.56
6	100 (+1)	15 (−1)	6.0 (+1)	4.72	15.81	20.53
7	60 (−1)	45 (+1)	6.0 (+1)	3.86	12.72	16.58
8	100 (+1)	45 (+1)	6.0 (+1)	4.81	15.91	20.72
9	47 (−1.67)	30 (0)	4.5 (0)	2.70	9.52	12.22
10	114 (+1.67)	30 (0)	4.5 (0)	2.91	10.35	13.26
11	80 (0)	4.8 (−1.67)	4.5 (0)	2.79	10.12	12.91
12	80 (0)	55.2 (+1.67)	4.5 (0)	3.89	12.88	16.77
13	80 (0)	30 (0)	2.0 (−1.67)	2.70	9.52	12.22
14	80 (0)	30 (0)	7.0 (+1.67)	4.62	15.87	20.49
15	80 (0)	30 (0)	4.5 (0)	2.97	10.51	13.48
16	80 (0)	30 (0)	4.5 (0)	3.32	11.17	14.48
17	80 (0)	30 (0)	4.5 (0)	3.27	11.23	14.50
18	80 (0)	30 (0)	4.5 (0)	2.92	10.19	13.11
19	80 (0)	30 (0)	4.5 (0)	3.13	10.89	14.01

**Table 2:** Central composite rotatable design (CCRD) for the independent variables considered for the UAE extraction with aqueous solutions of 1,2-propanediol from juçara fruit samples at 10 min, and the results obtained for the extraction assays in terms of yield of extraction of anthocyanins.

Assay	Amplitude (%)	C_alkanediol_ (wt%)	pH	C_cyanidin-3-O-glucoside_ (mg·g_dry biomass_^−1^)	C_cyanidin-3-O-rutinoside_ (mg·g_dry biomass_^−1^)	Yield of Extraction (mg_anthocyanins_·g_dry biomass_^−1^)
1	18 (−1)	15 (−1)	4.0 (−1)	7.64	36.64	44.29
2	42 (+1)	15 (−1)	4.0 (−1)	8.72	40.67	49.39
3	18 (−1)	45 (+1)	4.0 (−1)	8.72	38.94	47.66
4	42 (+1)	45 (+1)	4.0 (−1)	9.45	41.50	50.95
5	18 (−1)	15 (−1)	10.0 (+1)	7.64	35.66	43.30
6	42 (+1)	15 (−1)	10.0 (+1)	8.54	39.55	48.10
7	18 (−1)	45 (+1)	10.0 (+1)	7.63	35.89	43.52
8	42 (+1)	45 (+1)	10.0 (+1)	9.12	40.51	49.63
9	10 (−1.67)	30 (0)	7.0 (0)	7.21	34.47	41.68
10	50 (+1.67)	30 (0)	7.0 (0)	8.98	40.20	49.18
11	30 (0)	4.8 (−1.67)	7.0 (0)	8.13	39.69	47.82
12	30 (0)	55.2 (+1.67)	7.0 (0)	8.83	40.18	49.00
13	30 (0)	30 (0)	1.9 (−1.67)	8.79	39.14	47.93
14	30 (0)	30 (0)	12 (+1.67)	7.69	36.62	44.30
15	30 (0)	30 (0)	7.0 (0)	8.98	40.19	49.16
16	30 (0)	30 (0)	7.0 (0)	9.29	40.87	50.16
17	30 (0)	30 (0)	7.0 (0)	9.35	40.72	50.08
18	30 (0)	30 (0)	7.0 (0)	9.48	41.32	50.80
19	30 (0)	30 (0)	7.0 (0)	9.30	40.84	50.14

**Table 3:** Characterization of the juçara extracts obtained by UAE using different solvents in terms of yield of extraction, TPC, and ABTS and the corresponding standard deviation (s).

Solvent	Yield of Extraction ± s (mg_anthocyanins_·g_dry biomass_^−1^)	TPC ± s (mg_GAE_·g_dry biomass_^−1^)	ABTS ± s (μmol_TE_·g_dry biomass_^−1^)
Water	41.8 ± 0.7 ^e^	104.1 ± 0.8 ^d^	1163 ± 21 ^d^
1,2-ethanediol	49.60 ± 0.09 ^d^	107 ± 8 ^d^	1241 ± 8 ^bc^
1,2-propanediol	50 ± 1 ^cd^	117 ± 5 ^c^	1302 ± 32 ^a^
1,2-butanediol	51.0 ± 0.2 ^bcd^	130 ± 6 ^a^	1192 ± 16 ^cd^
1,2-pentanediol	52.63 ± 0.05 ^bc^	127 ± 4 ^ab^	1267 ± 35 ^ab^
1,2-hexanediol	55.5 ± 0.8 ^a^	126 ± 5 ^abc^	1302 ± 48 ^a^
Ethanol	49.0 ± 0.7 ^d^	120 ± 5 ^abc^	1229 ± 26 ^bc^
(1.0.1)	50 ± 1 ^d^	125 ± 11 ^abc^	1222 ± 13 ^bc^
(2.0.2)	49.8 ± 0.5 ^cd^	126 ± 1 ^abc^	1215 ± 25 ^bcd^
(2.0.0)	53.7 ± 0.4 ^ab^	119 ± 2 ^bc^	1255 ± 35 ^ab^

Different letters represent statistically different values (*p* ≤ 0.10), while the same letters represent statistically equal values (*p* > 0.10) by the Tukey test from ANOVA.

**Table 4:** Antioxidant activity by the ABTS method for soap bar and cream formulations.

Formulation	ABTS (μmol_TE_·g_formulation_^−1^)
Base bar (BB)	6.6 ± 0.6
Juçara bar (JB)	16.0 ± 0.5
Control Cream (CC)	0.10 ± 0.01
Juçara Cream (JC)	1.5 ± 0.1
Concentrated Juçara Cream (CJC)	3.3 ± 0.2

**Table 5:** List of compounds used in this work along with their CAS number, source, and purity.

Compound	CAS Number	Source	Purity (wt%)
1,2-ethanediol	107-21-1	Fisher Scientific (Geel, Belgium)	>99.0
1,2-propanediol	57-55-6	Sigma-Aldrich (Steinheim, Germany)	99.5
1,2-butanediol	584-03-2	Sigma-Aldrich (Steinheim, Germany)	>98.0
1,2-pentanediol	5343-92-0	TCI (Zwijndrecht, Belgium)	97.0
1,2-hexanediol	6920-22-5	Alfa Aesar (Kandel, Germany)	97.0
1,3-dimethoxypropan-2-ol (1.0.1)	623-69-8	Synthesized (Aveiro, Portugal)	>99.0
1,3-diethoxypropan-2-ol (2.0.2)	4043-59-8	Synthesized (Aveiro, Portugal)	>99.0
3-Ethoxypropane-1,2-diol (2.0.0)	1874-62-0	Synthesized (Aveiro, Portugal)	>99.0
2,2′-Azino-bis (3-ethylbenzothiazoline-6-sulfonic acid) diammonium salt (ABTS)	30931-67-0	Sigma-Aldrich (Steinheim, Germany)	98.0
Acetone	67-64-1	Fisher Scientific (Geel, Belgium)	>99.0
Acetonitrile	75-05-8	Fisher Scientific (Geel, Belgium)	99.9
Cyanidin 3-O-glucoside chloride	7084-24-4	Sigma-Aldrich (Steinheim, Germany)	>95.0
Cyanidin-3-O-rutinoside chloride	18719-76-1	Sigma-Aldrich (Steinheim, Germany)	>98.0
Ethanol	64-17-5	Fisher Scientific (Steinheim, Germany)	99.8
Folin–Ciocalteu reagent 2M	-	Panreac (Barcelona, Spain)	-
Gallic acid	149-91-7	Sigma-Aldrich (Steinheim, Germany)	99.5
Hydrochloric acid (HCl)	7647-01-0	Fisher Scientific (Steinheim, Germany)	37.0
Methanol	67-56-1	Fisher Scientific (Steinheim, Germany)	99.8
Potassium persulfate (K_2_S_2_O_8_)	7727-21-1	Scharlau (Barcelona, Spain)	99.0
Sodium carbonate (Na_2_CO_3_)	497-19-8	Prolabo (Geel, Belgium)	99.0
Sodium hydroxide (NaOH)	1310-73-2	Fisher Scientific (Steinheim, Germany)	98.0
Sulfuric acid (H_2_SO_4_)	7664-93-9	Sigma-Aldrich (Steinheim, Germany)	95.0
Trolox	53188-07-1	Acros Organics (Geel, Belgium)	97.0

## Data Availability

Not applicable.

## Supplementary Materials

The following supporting information can be downloaded at: https://www.mdpi.com/article/10.3390/molecules28041607/s1. Figure S1: Kinetic assays of juçara pulps at 10 MPa, 80 °C, and 1 mL.min^−1^ for PLE, and the identified mass-transfer mechanisms: CER—constant extraction rate period, FER—falling extraction rate period, DC—diffusion-controlled period; Figure S2: Pareto Chart of the CCRD (2^3^) regarding the yield of anthocyanins by the PLE method; Figure S3: Kinetic assays of juçara pulps at 30% of amplitude for UAE; Figure S4: Pareto Chart of the CCRD (2^3^) regarding the yield of anthocyanins by the UAE method; Figure S5: Chemical structure of the alkanediols and glycerol ethers used in this work as extractive solvents; Table S1: Regression coefficients and statistical parameters of the multiple regression of PLE responses in the central composite rotatable design (CCRD); Table S2: Regression coefficients and statistical parameters of the multiple regression adjustment of UAE responses in the central composite rotatable design (CCRD); Table S3: Temperature average for each different amplitude and the corresponding standard deviation (s) of the assays of the central composite rotatable design (CCRD) by UAE; Table S4: Initial pH (42.6 wt% of solvent) and final pH extract for different solvents by UAE.
