# Supplementary material for: Juçara Fruit (Euterpe Edulis Martius) Valorization Combining Emergent Extraction Technologies and Aqueous Solutions of Alkanediols
Source: Molecules. 2023 Feb 7;28(4):1607. doi: 10.3390/molecules28041607 (PMC9966649; doi:10.3390/molecules28041607)
Supplement: Supplementary file 1 [file molecules-28-01607-s001.zip › molecules-2173701-supplementary.pdf]

Supplementary Materials

Article

Juçara Fruit (*Euterpe edulis* Martius) Valorization Combining Emergent Extraction Technologies and Aqueous Solutions of Alkanediols

Bruna P. Soares [1,2], Ana M. Ferreira [1], Marina Justi [1], Luiz Gustavo Gonçalves Rodrigues [2], J. Vladimir Oliveira [2], Simão P. Pinho [3,4,*] and João A. P. Coutinho [1]

[1] CICECO—Aveiro Institute of Materials, Department of Chemistry, University of Aveiro, 3810-193 Aveiro, Portugal
[2] Laboratory of Thermodynamics and Supercritical Technology (LATESC), Department of Chemical and Food Engineering (EQA), Federal University of Santa Catarina (UFSC), Florianópolis 88040-900, Brazil
[3] Centro de Investigação de Montanha (CIMO), Instituto Politécnico de Bragança, Campus de Santa Apolónia, 5300-253 Bragança, Portugal
[4] Laboratório para a Sustentabilidade e Tecnologia em Regiões de Montanha, Instituto Politécnico de Bragança, Campus de Santa Apolónia, 5300-253 Bragança, Portugal
* Correspondence: spinho@ipb.pt

Figures

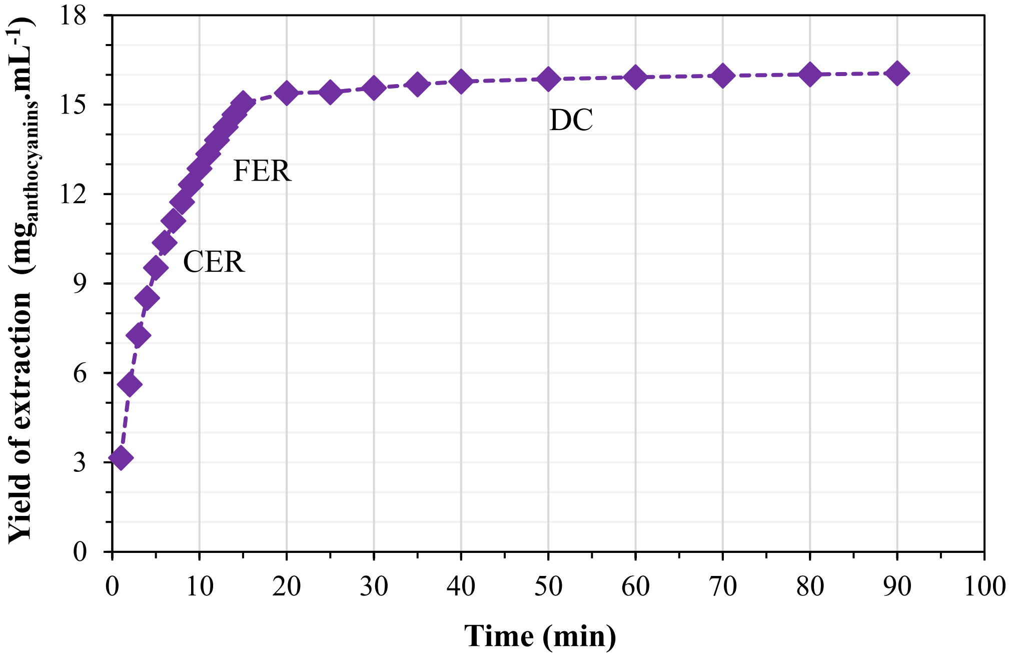

Figure S1. Kinetics assays of juçara pulps at 10 MPa, 80°C and 1 mL.min^{-1} for PLE and the identified mass-transfer mechanisms: CER - constant extraction rate period, FER - falling extraction rate period, DC - diffusion-controlled period.

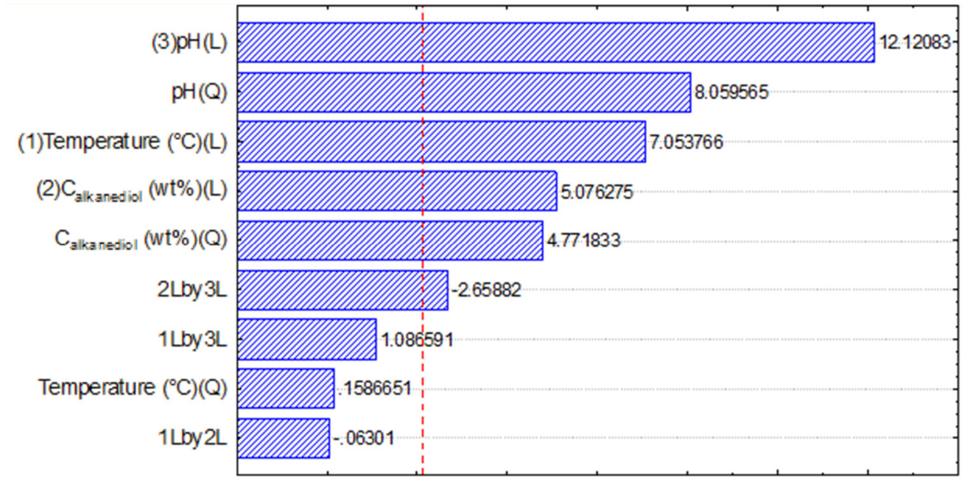

Figure S2: Pareto Chart of the CCRD (2^3) regarding the yield of anthocyanins by PLE method.

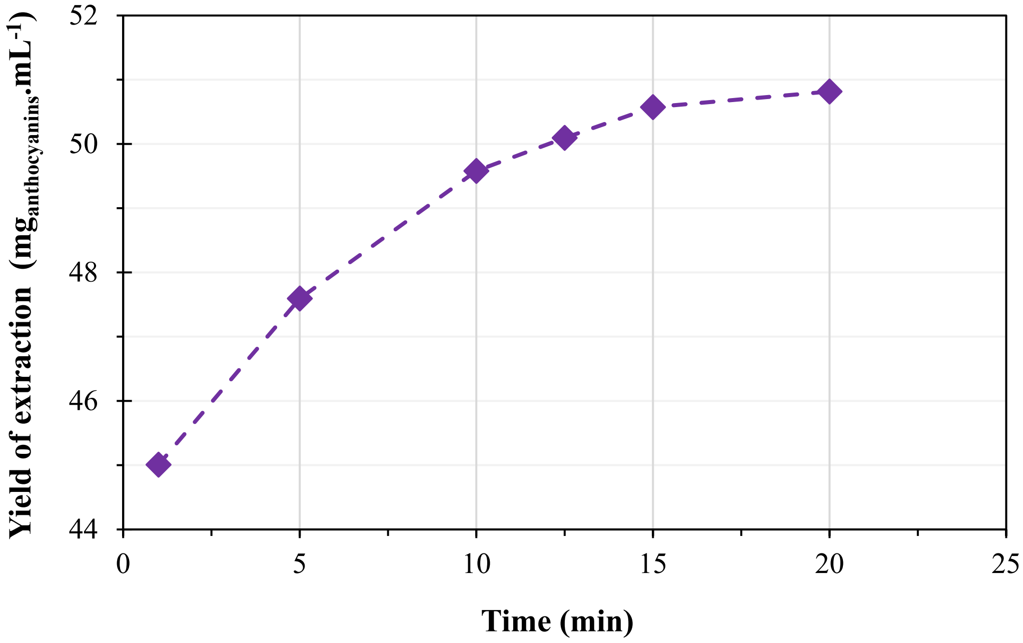

Figure S3: Kinetics assays of juçara pulps at 30% of amplitude for UAE.

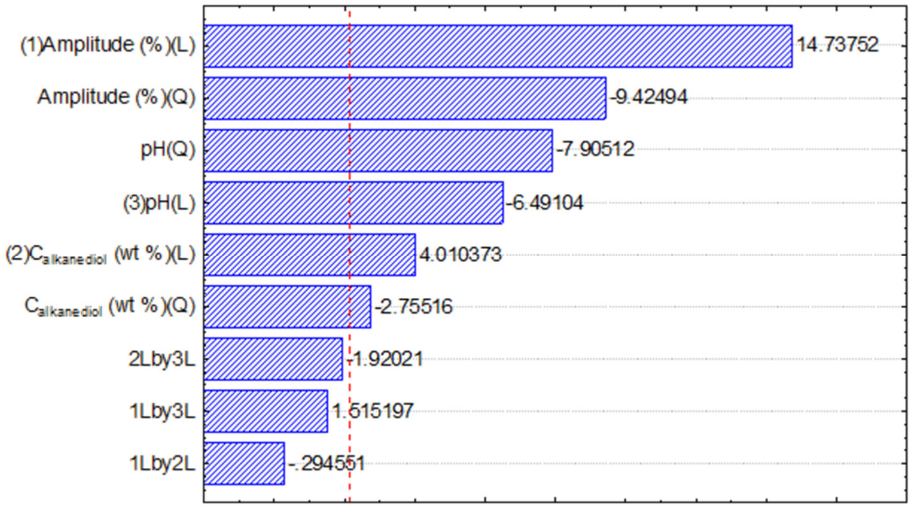

Figure S4: Pareto Chart of the CCRD (2^3) regarding the yield of anthocyanins by UAE method.

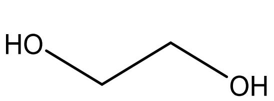

1,2-Ethanediol

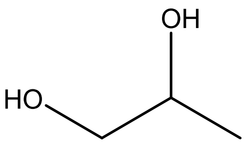

1,2-Propanediol

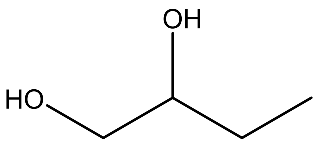

1,2-Butanediol

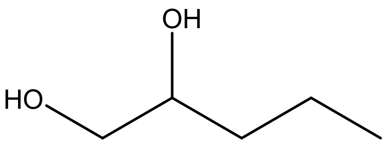

1,2-Pentanediol

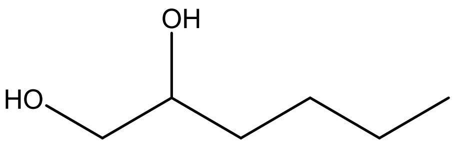

1,2-Hexanediol

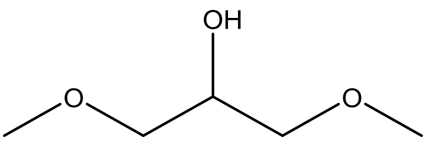

1,3-dimethoxypropan-2-ol [1.0.1]

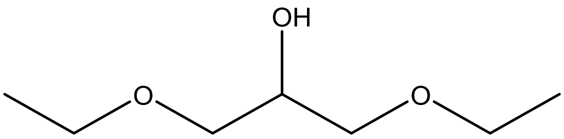

1,3-diethoxypropan-2-ol [2.0.2]

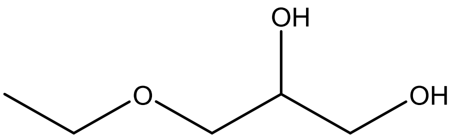

3-Ethoxypropane-1,2-diol [2.0.0]

Figure S5: Chemical structure of the alkanediols and glycerol ethers used in this work as extractive solvents.

Tables

Table S1: Regression coefficients and statistical parameters of the multiple regression of PLE responses in the Central Composite Rotatable Design (CCRD).

Response variables	Factors	Regression coefficient	Standard error	t-value	p-value	Lower limit	Upper limit
Yield of extraction of anthocyanins	Constant	12.38716	1.668534	7.42398	0.001757	8.83010	15.94421
($mg_{anthocyanins}.g_{dry\ biomass}^{-1}$)	x_1	0.05881	0.008330	7.05959	0.002124	0.04105	0.07656
	x_2^2	0.00320	0.000523	6.10938	0.003633	0.00208	0.00431
	x_3	-3.14282	0.689633	-4.55723	0.010361	-4.61301	-1.67263
	x_3^2	0.59853	0.073937	8.09514	0.001266	0.44091	0.75615
	$x_2.x_3$	-0.02983	0.006971	-4.27947	0.012853	-0.04469	-0.01497
R^2	0.81						
adjusted R^2	0.74						
$F_{calculated}$	26.99						
p-value	$1.89*10^{-6}$						
p_{model}	< 0.1						

Note – x_1: temperature (°C); x_2: $C_{alkanediol}$ (wt%); x_3: pH. Confidence level 90%.

Table S2. Regression coefficients and statistical parameters of multi regression adjustment of UAE responses in the Central Composite Rotatable Design (CCRD).

Response variables	Factors	Regression coefficient	Standard error	t-value	p-value	Lower limit	Upper limit
Yield of extraction of anthocyanins ($mg_{anthocyanins}.g_{dry\ biomass}^{-1}$)	Constant	27.25774	1.570059	17.36097	0.000065	23.91062	30.60487
	x_1	0.82742	0.068345	12.10644	0.000267	0.68172	0.97312
	x_1^2	-0.01053	0.001117	-9.42494	0.000707	-0.01291	-0.00815
	x_2	0.15912	0.043657	3.64482	0.021871	0.06605	0.25219
	x_2^2	-0.00194	0.000706	-2.75516	0.051103	-0.00345	-0.00044
	x_3	1.60348	0.250899	6.39095	0.003077	1.06860	2.13836
	x_3^2	-0.13904	0.017589	-7.90512	0.001385	-0.17654	-0.10155
R^2	0.95						
adjusted R^2	0.93						
$F_{calculated}$	30.26						
p-value	$9.36*10^{-7}$						
p_{model}	< 0.1						

Note – x_1: amplitude (%); x_2: $C_{alkanediol}$ (wt %); x_3: pH. Confidence level 90%.

Table S3: Temperature average for each different amplitude and the corresponding standard deviation (s) of the assays of the Central Composite Rotatable Design (CCRD) by UAE.

Amplitude (%)	Final temperature average (°C) ± s
10	67.8
18	92 ± 4
30	93 ± 2
42	97 ± 3
50	99.1

Table S4: Initial pH (42.6 wt.% of solvent) and final extract pH for different solvents by UAE.

Solvent	Initial solvent/water mixture pH (before adjusting)	Final extract pH
Water	5.65	4.12
1,2-ethanediol	6.44	4.35
1,2-propanediol	6.88	4.49
1,2-butanediol	6.63	4.47
1,2-pentanediol	6.30	4.45
1,2-hexanediol	6.22	4.31
Ethanol	7.28	4.53
[1.0.1]	3.75	4.82
[2.0.2]	3.60	4.72
[2.0.0]	7.67	4.56
